# Supplementary material for: Genetic and morphological estimates of androgen exposure predict social deficits in multiple neurodevelopmental disorder cohorts
Source: Mol Autism. 2021 Jun 9;12:43. doi: 10.1186/s13229-021-00450-w (PMC8190870; doi:10.1186/s13229-021-00450-w)
Supplement: Supplementary file 2 — Additional file 2. Figure 1. Facial landmarking by machine learning. Figure 2. Choosing the optimal number of factors in devGenes parent-report data. Figure 3. Uniquenesses of individual SCQ items in the SPARK factor analysis (8 factors). Figure 4. Null distributions and empirical p-values for 2D:4D ratio and facial masculinity. [file 13229_2021_450_MOESM2_ESM.pdf]

1 Supplementary Figures

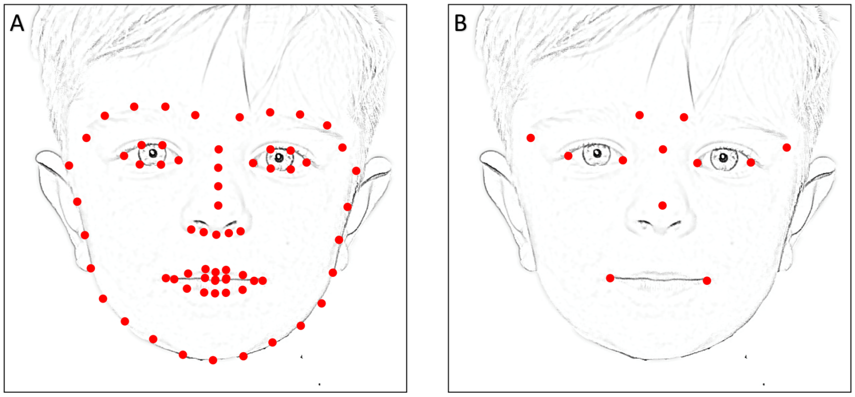

Figure 1: **Facial landmarking by machine learning** (A) The annotation of 68 facial coordinates by *dlib*. (B) After filtering the redundant points, 12 landmarks were chosen.

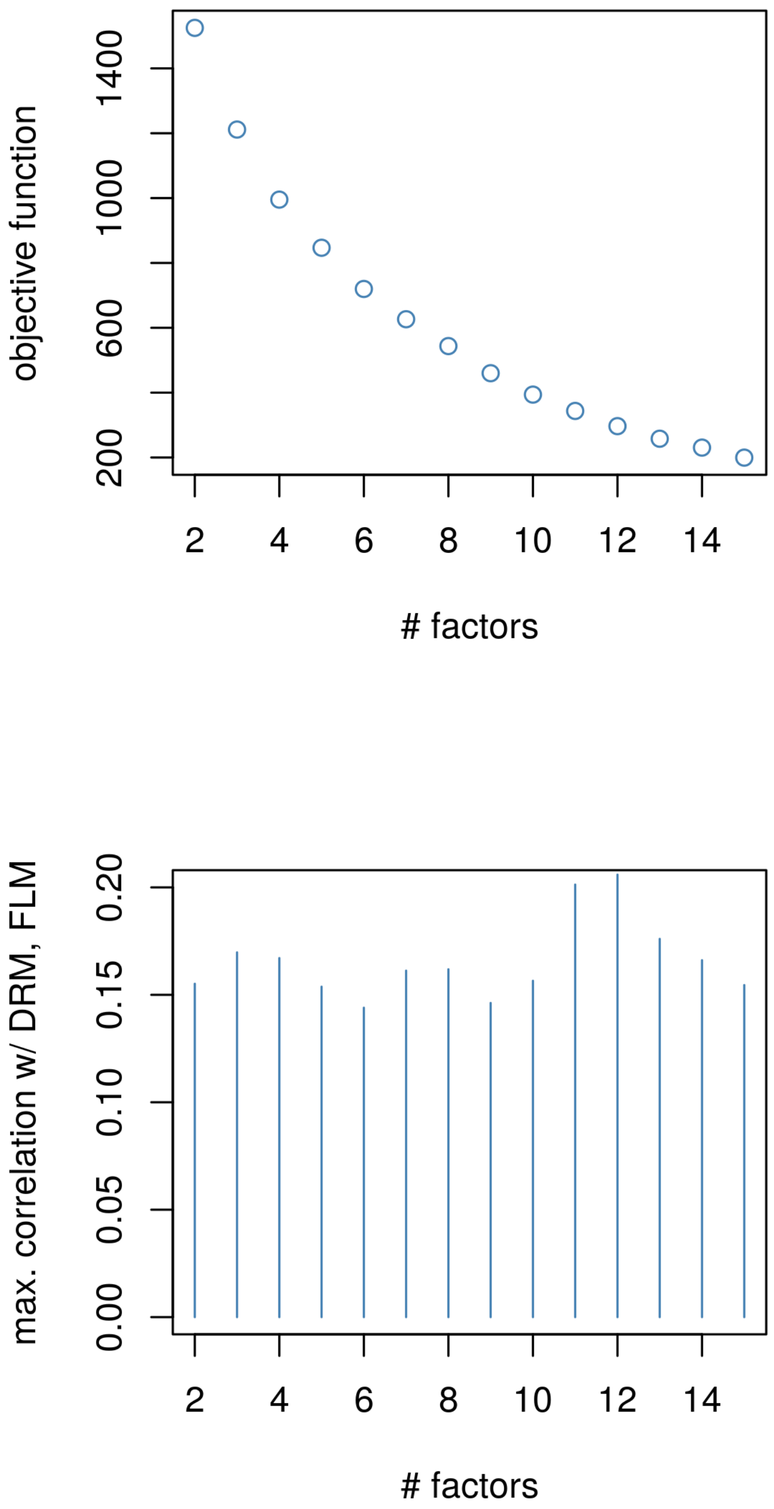

Figure 2: **Choosing the optimal number of factors in devGenes parent-report data.** The test statistic for the factor model from two to 15 factors (top). The maximal correlation coefficient among factor scores with DRM and FLM, as a function of the number of factors (bottom).

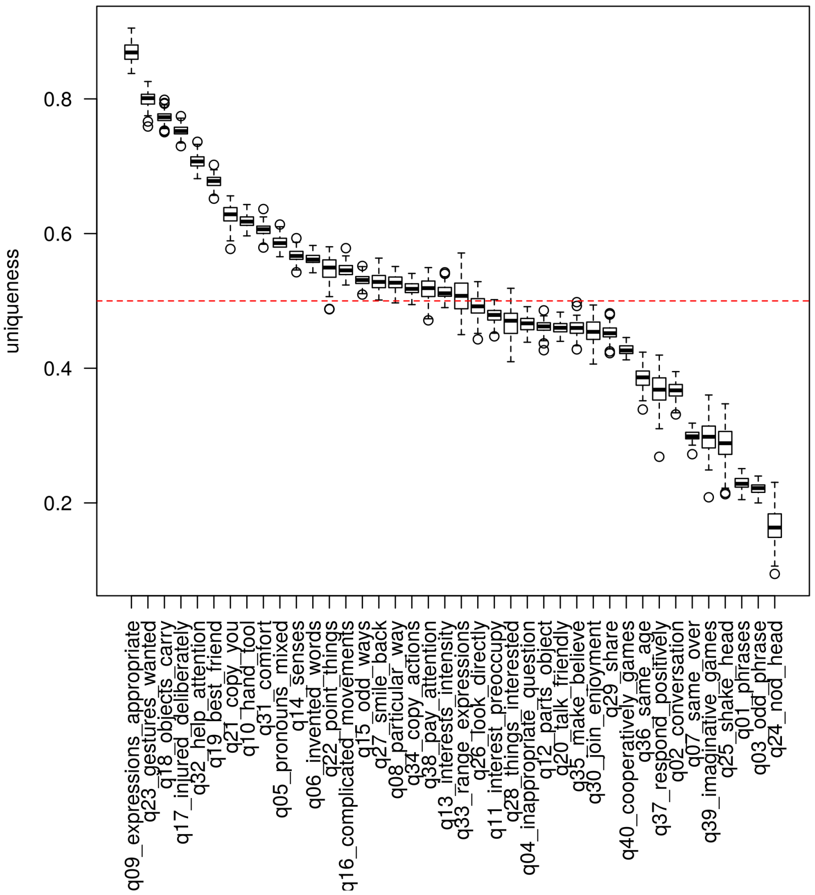

Figure 3: **Uniquenesses of individual SCQ items in the SPARK factor analysis (8 factors).** Factor analyses using eight factors were repeatedly performed on bootstrap samples of the data (100 bootstrap samples), and the uniqueness for each item was recorded in each permutation. Items with 50% uniqueness were considered separately from the factors in the PRS associations.

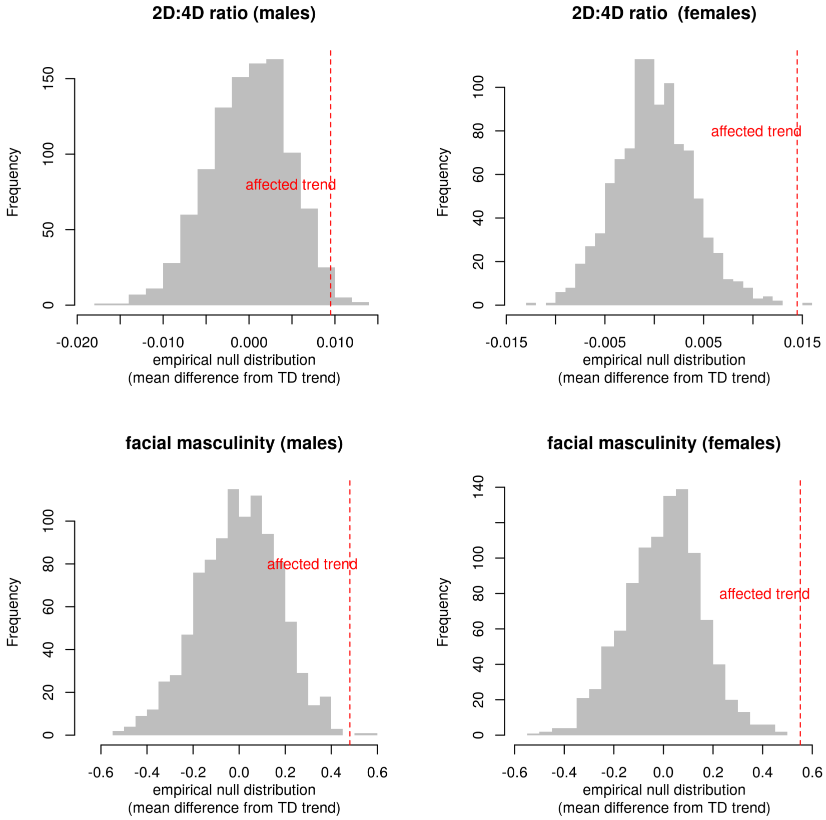

Figure 4: **Null distributions and empirical p-values for 2D:4D ratio and facial masculinity.** The mean difference (over age) from bootstrapped lowess trends of typically-developing individuals (TD) relative to the mean TD trend constitutes the null distribution. The mean difference of the ”affected” trend for 2D:4D ratio (males and females) and facial masculinity (males and females) is shown in red. Note that because *decreasing* 2D:4D ratio corresponds to increased digital masculinity, an extreme positive value corresponds to a failure to reject the null hypothesis under consideration: $M_{NDD} \leq M_{TD}$ (where M is some objective measure of masculinity). The corresponding empirical p-values for 2D:4D ratio for males and females are $p = 0.988$ and $p = 0.999$, respectively. In contrast, for facial masculinity, whose values are positively correlated to masculinity, the null hypothesis is rejected ($p = 0.002$ and $p < 0.001$, for males and females, respectively). Compare with Figure 2, panels C, D and G, H in the main text.
